# Synbiotic of *Pediococcus acidilactici* and Inulin Ameliorates Dextran Sulfate Sodium-Induced Acute Ulcerative Colitis in Mice

**DOI:** 10.4014/jmb.2308.08056

**Published:** 2023-12-27

**Authors:** Mingzhu Wang, Longzhou Zhang, Huiyan Piao, Yuanming Jin, Chengdu Cui, Xin Jin, Lianhua Cui, Chunri Yan

**Affiliations:** 1 Department of Animal Science, Yanbian University, Yanji, Jilin 133002, P.R. China; 2 Engineering Research Center of North-East Cold Region Beef Cattle Science & Technology Innovation, Ministry of Education, Yanbian University, Yanji, Jilin 133002, P.R. China; 3 Department of Chemistry, National Demonstration Centre for Experimental Chemistry Education, Yanbian University, Yanji, Jilin 133002, P.R. China; 4 Department of Animal Medicine, Yanbian University, Yanji, Jilin 133002, P.R. China; 5 Laboratory Animal Center?Yanbian University, Yanji, Jilin 133002, P.R. China; 6 Department of Preventive Medicine, Medical College, Yanbian University, Yanji, Jilin 133002, P.R. China

**Keywords:** *Pediococcus acidilactici*, inulin, synbiotics, ulcerative colitis, mice

## Abstract

Colitis is a major gastrointestinal disease that threatens human health. In this study, a synbiotic composed of inulin and *Pediococcus acidilactici* (*P. acidilactici*) was investigated for its ability to alleviate dextran sulfate sodium (DSS)-induced colitis. The results revealed that the synbiotic, composed of inulin and *P. acidilactici*, attenuated the body weight loss and disease activity index (DAI) score in mice with DSS-mediated colitis. Determination of biochemical indicators found that the synbiotic increased anti-oxidation and alleviated inflammation in mice. Additionally, histopathological examination revealed that colonic goblet cell loss and severe mucosal damage in the model group were significantly reversed by the combination of inulin and *P. acidilactici*. Moreover, synbiotic treatment significantly reduced the levels of IL-1β, TNF-α, and IL-6 in the serum of mice. Thus, a synbiotic composed of inulin and *P. acidilactici* has preventive and therapeutic effects on DSSinduced colitis in mice.

## Introduction

In previous feeding models, newborn animals with diarrhea were frequently administered antibiotics or vaccines as a preventive measure against the occurrence of this condition. However, the widespread and indiscriminate use of antibiotics has resulted in a range of emerging issues, including bacterial resistance, residues of veterinary drugs, and disruption of the microecological balance [1, 2]. Therefore, it is imperative that environmentally friendly and health-conscious alternatives to antibiotics be identified. Zhongfeng Yuan *et al*. [3] demonstrated that *P. acidilactici* effectively restored intestinal flora dysbiosis induced by antibiotics in mice while promoting the proliferation of beneficial bacteria through this restoration process. These beneficial bacteria exerted inhibitory effects on pathogenic bacteria growth via ecological occupancy and colonization resistance mechanisms while also regulating host immune function through the production of metabolites, such as lactic acid and short-chain fatty acids. Additionally, inulin was employed as a prebiotic agent to facilitate probiotic proliferation and maintain enteric microecological balance alongside host well-being, thereby presenting an innovative strategy for managing diarrhea in young animals [4].

In this study, we administered synbiotics with *P. acidilactici* and varying ratios of inulin intragastrically for a prophylactic period of 14 days and continued during the modeling period. Finally, by analyzing the disease phenotype indicators, oxidative stress indicators, inflammatory cytokine expression, and organic acid levels in mice [5], we further studied the regulatory effects of *P. acidilactici*-inulin synbiotic on intestinal inflammation and screened out the optimal ratio of inulin to provide an experimental basis for solving the problem of diarrhea in newborn animals.

## Materials and Methods

### Animal Care

The animal experiments involved in this study strictly followed the requirements of the Guidelines for the Ethics and Use of Agricultural Animals in Research and Teaching [6] and were reviewed by the Experimental Animal Ethics Committee of Yanbian University (ethical review acceptance no. YD20220718003).

### Materials

All mice used in the experiment were SPF C57BL/6 (male, 6–8 weeks old, weighing 18–22 g) purchased from the Experimental Animal Center of Yanbian University. Inulin was purchased from Baiyao Biotechnology (China). *P. acidilactici* was obtained from the Functional Feed Additives Research Laboratory, College of Agriculture, Yanbian University.

### Feeding and Management

The mice were reared in the laboratory animal center (SPF environment), and the environmental conditions of the mice room were maintained at 23 ± 2°C, with indoor-air relative humidity of 55–65%, and a 12 h/12 h day/night cycle. The animals were housed in cages that provided independent ventilation, standard mice diet, and free access to water and food. The entire experiment was carried as per the rules and regulations of the laboratory animal center.

### Animal Model Establishment

**Fig. 1:**
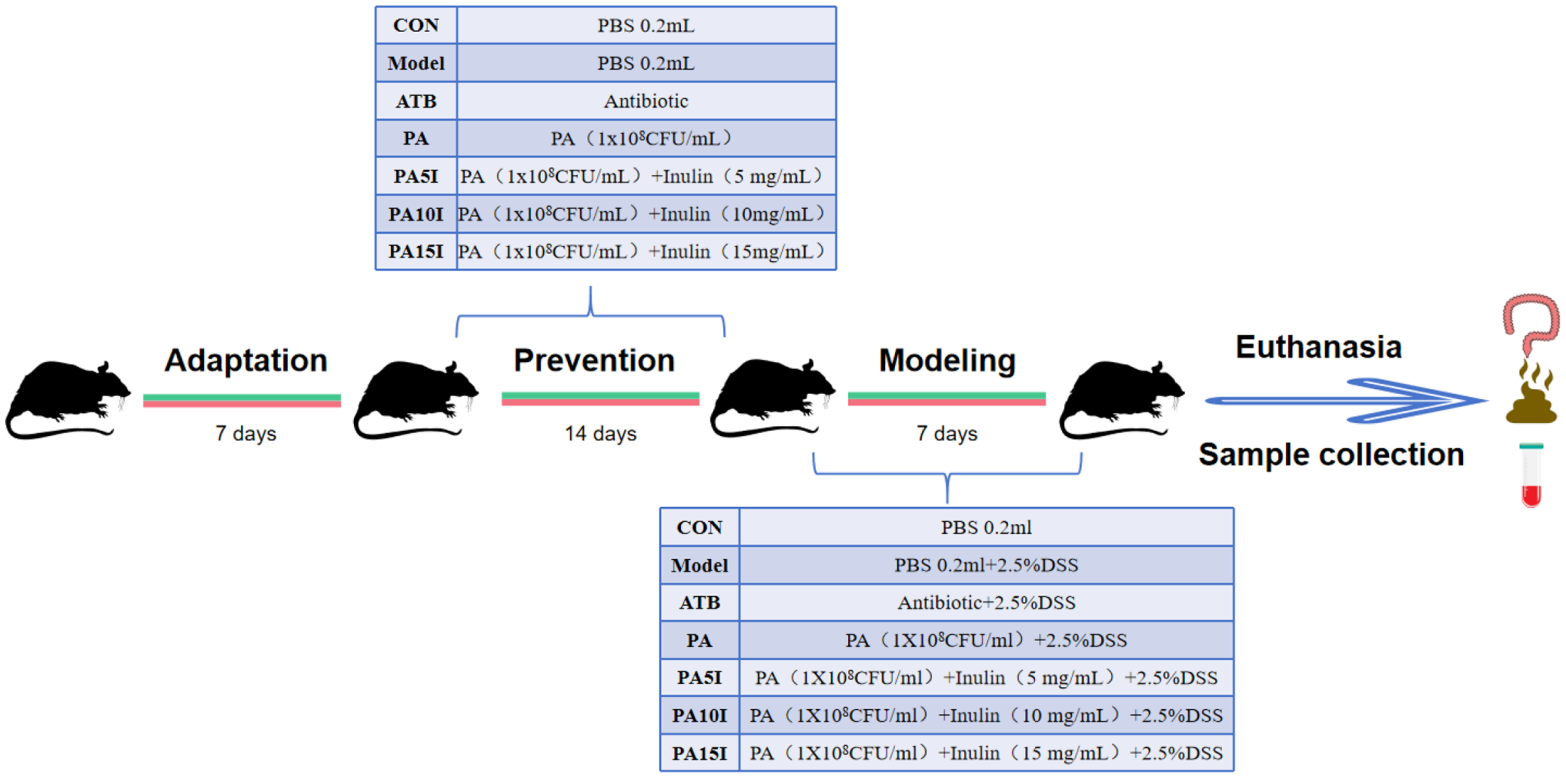
Trial group design and do sing regimen. CON: PBS; Model: PBS+2.5%DSS; ATB: Antibiotic+2.5%DSS; PA: *P. acidilactici*+2.5%DSS; PA5I: *P. acidilactici*+ Inulin (5 mg/ml)+2.5%DSS; PA10I: *P. acidilactici*+Inulin (10 mg/ml)+2.5%DSS; PA15I: *P. acidilactici*+Inulin (15 mg/ml)+2.5%DSS

Seventy mice were fed and watered ad libitum and pre-housed for 7 days. On the first day of the formal experiment, the 70 mice were randomly divided into seven groups, with 10 mice in each group: the control (CON), model (Model), antibiotic (ATB), *P. acidilactici* (PA), low-dose inulin (PA5I), medium-dose inulin (PA10I), and high-dose inulin (PA15I) groups. The entire experiment duration was divided into a prevention period (14 days) and modeling period (7 days). The specific treatments are described in Fig. 1.

### Measurement Indices and Methods

**Daily Mice Observation**

During the prevention and modeling periods, gavage was performed according to the experimental grouping and drug administration scheme. The body weight and food and water intakes of the mice were recorded accurately every day, and sufficient water and feed were provided for each cage. During the modeling period, hair color, mental state, fecal characteristics, and hematochezia of the mice were observed to assess the DAI index scores.

### Mice Sample Collection

At the end of the last gavage, feeding was stopped, and sampling was performed after 24 h of fasting. Sterile ophthalmic scissors, forceps, and EP tubes were prepared in advance. Feces were collected before dissection, and transferred to an EP tube with forceps, marked, and stored at -80°C for subsequent microbial colony counting. After the euthanasia, blood samples of the eyeball were collected and rested at a low temperature until stratification occurred. Following centrifugation at 3,500 ×*g* and 4°C for 10 min (5910R; Eppendorf, Germany), the supernatant was stored at −20°C until further use. After dissection, the thymus, spleen, and colon were collected to accurately record the weight of organs and colon length. Approximately 1 cm of the middle colon was excised and fixed in 4% paraformaldehyde. Cecal contents were collected and instantly placed in liquid nitrogen and transferred to a refrigerator for storage at −80°C.

### Mice Disease Activity Index

The scoring standard of the mice disease activity index (DAI) was comprehensively evaluated based on three aspects reflecting the health condition of the mice to a certain extent [7]. During the experiment, changes in body weight, fecal traits, hematochezia, and the mental state of the mice were carefully recorded daily. Fecal occult blood was detected using the fecal occult blood reagent of the piramido method (Shanghai Yaji Biotechnology Co., Ltd., China) and the specific operation was carried out according to the instructions included with the reagent. The presence of red-brown or bright red blood in stool indicated bloody stool, while the normal feces of mice were formed and granular. If there was increased viscosity and easy dispersion without adherence to the anus, it was considered loose stool.

The mice were scored as shown in Table 1:

DAI = (weight score + stool score + hematochezia score) /3 [8].

### Immune-Organ Indices of Mice

The mice immune-organ indices were calculated using the following formula [9]:

Spleen index = spleen weight (g)/final body weight (g) ×10, which is the weight of the mice spleen per 10 grams of body weight.

Thymus index = thymus weight (g)/final body weight (g) ×10, which is the weight of the mice thymus per 10 grams of body weight.

### Hematoxylin-Eosin (HE) Staining of the Colon Tissue of Mice

After the samples were collected, approximately 1 cm of the middle colon tissue was excised and fixed with 4%paraformaldehyde solution for more than 24 h. Subsequently, the colon tissue was removed for trimming, and the mesentery and other attachments were removed. Then, dehydration, routine paraffin embedding, and HE staining were started.

### Blood Indicators

A kit (Nanjing Institute of Bioengineering, China) was used to determine the amounts of malonaldehyde (MDA), myeloperoxidase (MPO), nitric oxide synthase (NOS), superoxide dismutase (SOD), glutathione peroxidase (GSH-Px), and total antioxidant capacity (T-AOC) [10]. In addition, the levels of TNF-α, IL-6, IL-1β, and other serum inflammatory cytokines were determined by using a kit (Shanghai Enzyme Biotechnology Co., Ltd., China).

### Statistical Analysis

SPSS 17.0 software (IBM, USA) was used for statistical analysis; one-way ANOVA was used for analysis of variance. All data were expressed as mean ± SD and differences were considered statistically significant at *p*<0.05.

## Results

### Effects on Daily Weight Gain

**Fig. 2:**
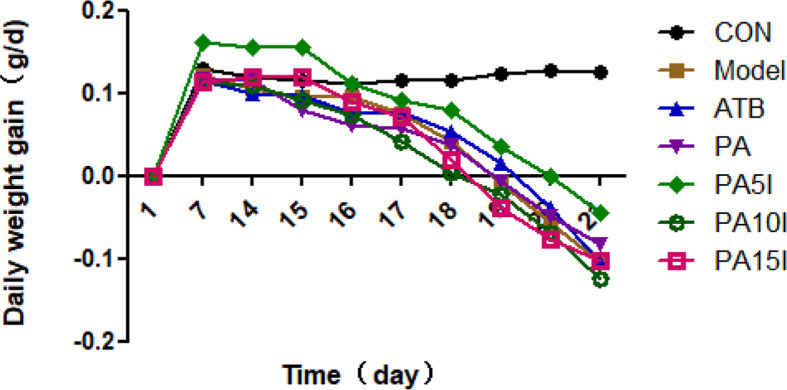
Changes of daily weight gain in mice. CON: PBS; Model: PBS+2.5%DSS; ATB: Antibiotic+2.5%DSS; PA: *P. acidilactici*+2.5%DSS; PA5I: *P. acidilactici*+ Inulin (5 mg/ml)+2.5%DSS; PA10I: *P. acidilactici*+Inulin (10 mg/ml)+2.5%DSS; PA15I: *P. acidilactici*+Inulin (15 mg/ml)+2.5%DSS

As shown in Fig. 2, the test results from days 1 to 14 showed that the daily weight gain of mice in the four treatment groups containing *P. acidilactici* was promoted in comparison with the other three groups. The PA5I group gained the most weight (average daily weight gain, 2.17 g/mouse), whereas the ATB group gained the least (average daily weight gain, 1.39 g/mouse). Moreover, the results showed that the daily weight gain of the PA5I group was significantly higher than that of the CON group (1.50 g), Model group (1.55 g), ATB group (1.39 g), and PA group (1.62 g) (*p* < 0.05). Meanwhile, there was no significant difference between the other groups and CON group (*p* > 0.05).

The data from days 14 to 21 showed that compared with the CON group, the daily weight gain of the other groups decreased. Specifically, the Model group experienced weight loss during the modeling phase on the third day, while the PA5I group exhibited weight loss during the modeling phase on the fifth day. By the end of 21 days, we observed that the daily weight gain of PA5I group was significantly higher than that of Model group, ATB group, PA10I group, and PA15I group (*p* < 0.05), and there was no significant difference between PA5I group and PA group (*p* > 0.05). Overall, the mice treated with PA and 5 mg of inulin showed lesser weight loss than the mice in other model groups.

### Effect on DAI Score

**Fig. 3:**
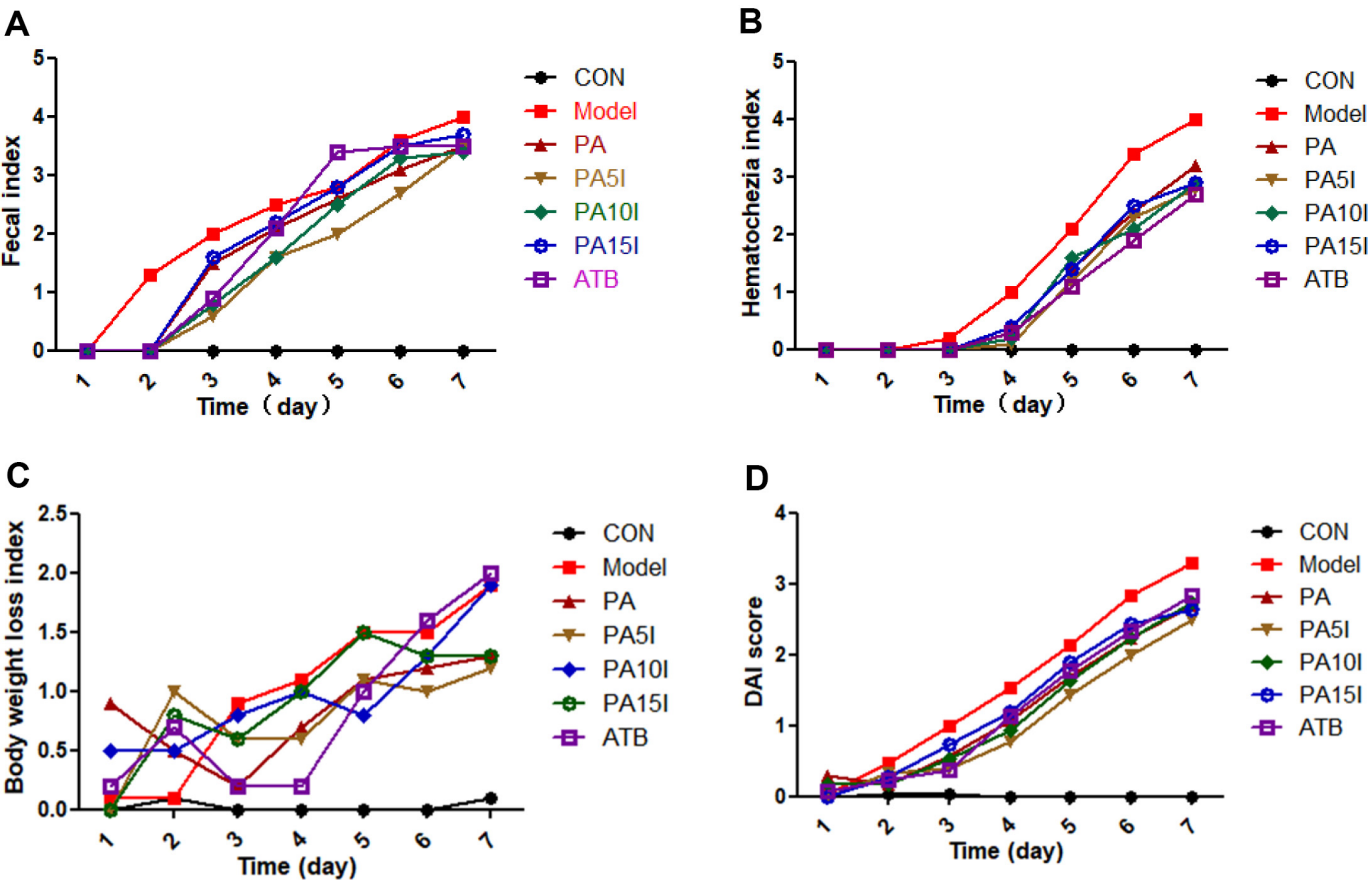
Changes in growth performance of mice. CON: PBS; Model: PBS+2.5%DSS; ATB: Antibiotic+2.5%DSS; PA: *P. acidilactici*+2.5%DSS; PA5I: *P. acidilactici*+Inulin (5 mg/ml)+2.5%DSS; PA10I: *P. acidilactici*+Inulin (10 mg/ml)+2.5%DSS; PA15I: *P. acidilactici*+Inulin (15 mg/ml)+2.5%DSS Note: (**A**) Fecal index (**B**) Hematochezia index (**C**) Body weight loss index (**D**) DAI score

The effects of inulin combined with *P. acidilactici* on the stool index, hematochezia index, weight loss index, and DAI score of mice are shown in Fig. 3A-3D. Throughout the experimental period, the stool index, hematochezia index, weight loss index, and DAI score of the CON group remained stable, indicating satisfactory growth and development without any adverse manifestations. On the second day of testing, all indices significantly increased in the Model group. However, we observed that the aforementioned indices in the experimental groups generally displayed lower values than those of the Model group. Notably, both PA5I and PA10I groups demonstrated significantly lower fecal indices than that observed in the Model group (*p* < 0.05). In the weight loss index, the Model group, ATB group and PA10I group were significantly higher than other groups (*p* < 0.05). The PA5I group displayed the lowest value for DAI index with superior efficacy when compared to other groups.

### Effects on Immune-Organ Index

**Fig. 4:**
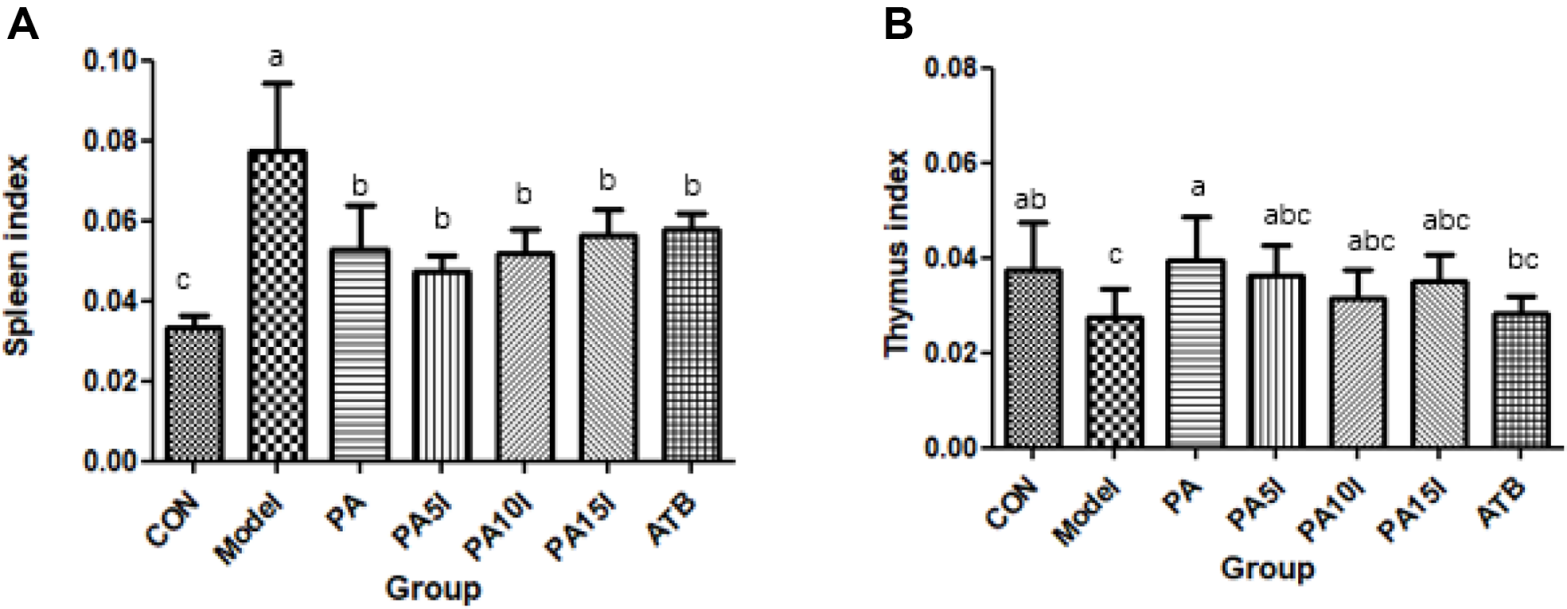
Changes of immune organ index in mice. CON: PBS; Model: PBS+2.5%DSS; ATB: Antibiotic+2.5%DSS; PA: *P. acidilactici*+2.5 %DSS; PA5I: *P. acidilactici*+Inulin (5 mg/ml)+2.5%DSS; PA10I: *P. acidilactici*+Inulin (10 mg/ml)+2.5%DSS; PA15I: *P. acidilactici*+Inulin (15 mg/ml)+2.5%DSS Note: (**A**) Spleen index (**B**) Thymus index Different letters above the histogram indicate significant differences (*p* < 0.05).

The effect of inulin combined with *P. acidilactici* on the immune-organ index of mice is shown in Fig. 4A and 4B. The spleen index of mice in the Model group exhibited a significant increase (*p* < 0.05) while the thymus index showed a significant decrease (*p* < 0.05), indicating that the mice in the Model group experienced pronounced immune damage compared to those in the CON group. After intragastric administration, there was a notable reduction in the spleen index within each treatment group, which was significantly lower than that of the Model group (*p* < 0.05). Conversely, the thymus index rebounded, with the PA group demonstrating a significantly higher value than that of the Model group (*p* < 0.05).

### Effects on Colon Morphology and Length

**Fig. 5:**
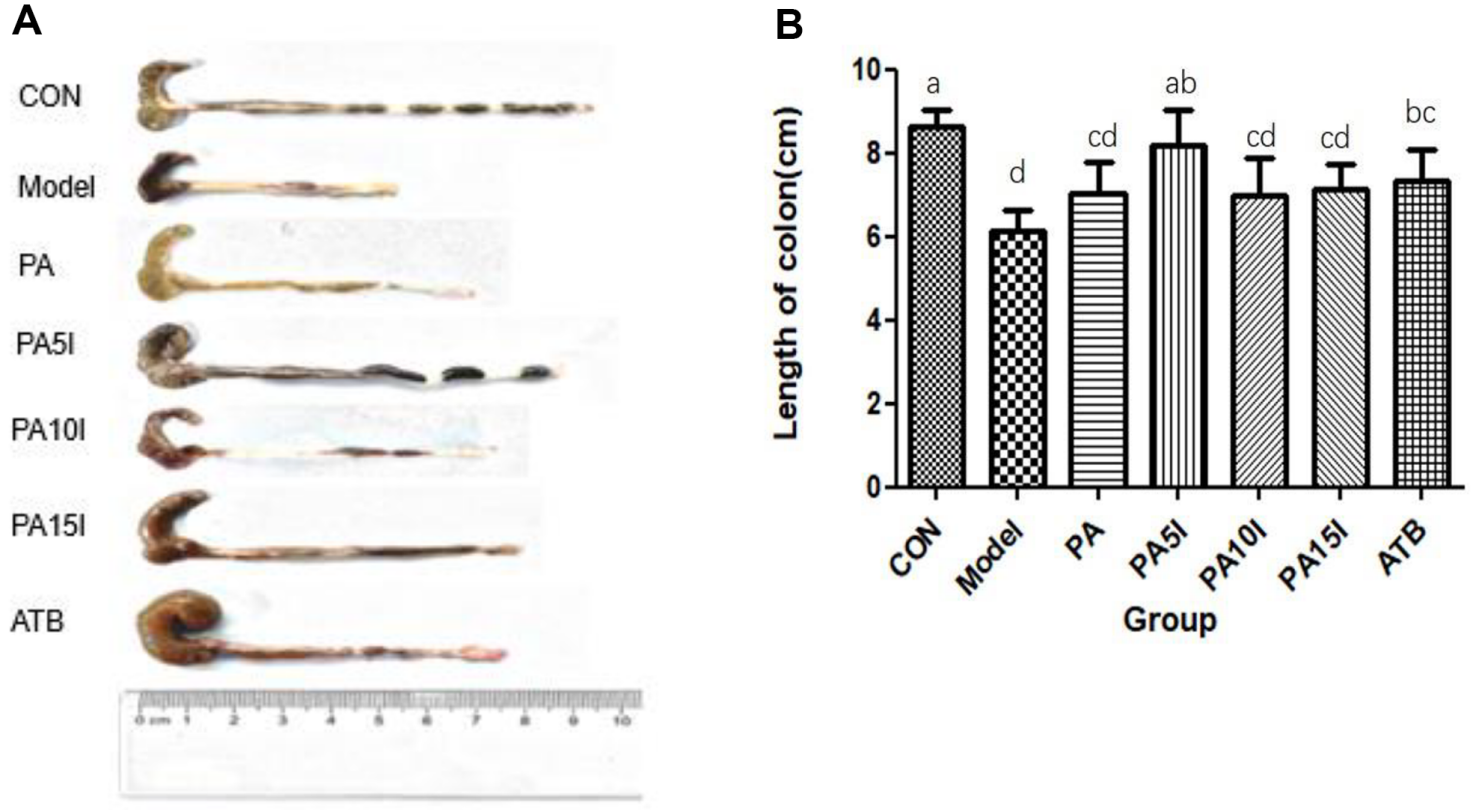
Colonic changes in mice. CON: PBS; Model: PBS+2.5%DSS; ATB: Antibiotic+2.5%DSS; PA: *P. acidilactici*+2.5 %DSS; PA5I: *P. acidilactici*+Inulin (5 mg/ml)+2.5%DSS; PA10I: *P. acidilactici*+Inulin (10 mg/ml)+2.5%DSS; PA15I: *P. acidilactici*+Inulin (15 mg/ml)+2.5%DSS Note: (**A**) Morphological change (**B**) Length change Different letters above the histogram indicate significant differences (*p* < 0.05).

Fig. 5A and 5B illustrates the morphological and length alterations observed in the colons of mice. In the CON group, the colon exhibited intact morphology, normal length, regular colonic contents, and feces at its distal end. Conversely, in the Model group, which experienced pronounced DSS-induced effects, a significant reduction in colon length was evident when compared to the CON, PA5I, and ATB groups (*p* < 0.05). Furthermore, severe inflammation led to hematochezia resulting in complete lack of form in colonic contents for this group. The colon length in the PA5I group and ATB group did not show a significant difference compared to the CON group (*p* > 0.05), but it was significantly different from other groups (*p* < 0.05). There was no significant variation in colon length among the PA, PA10I, PA15I, and Model groups (*p* > 0.05). The colon morphology of the PA5I group exhibited improved shortening of the colon, formation of colonic contents (although not granular), and substantial amelioration of hematochezia. However, distal colon atrophy and mild hematochezia were observed in the ATB group. Consequently, the PA5I group demonstrated less intestinal damage and proved superior to the ATB group.

### Effect on Colonic Pathology

**Fig. 6:**
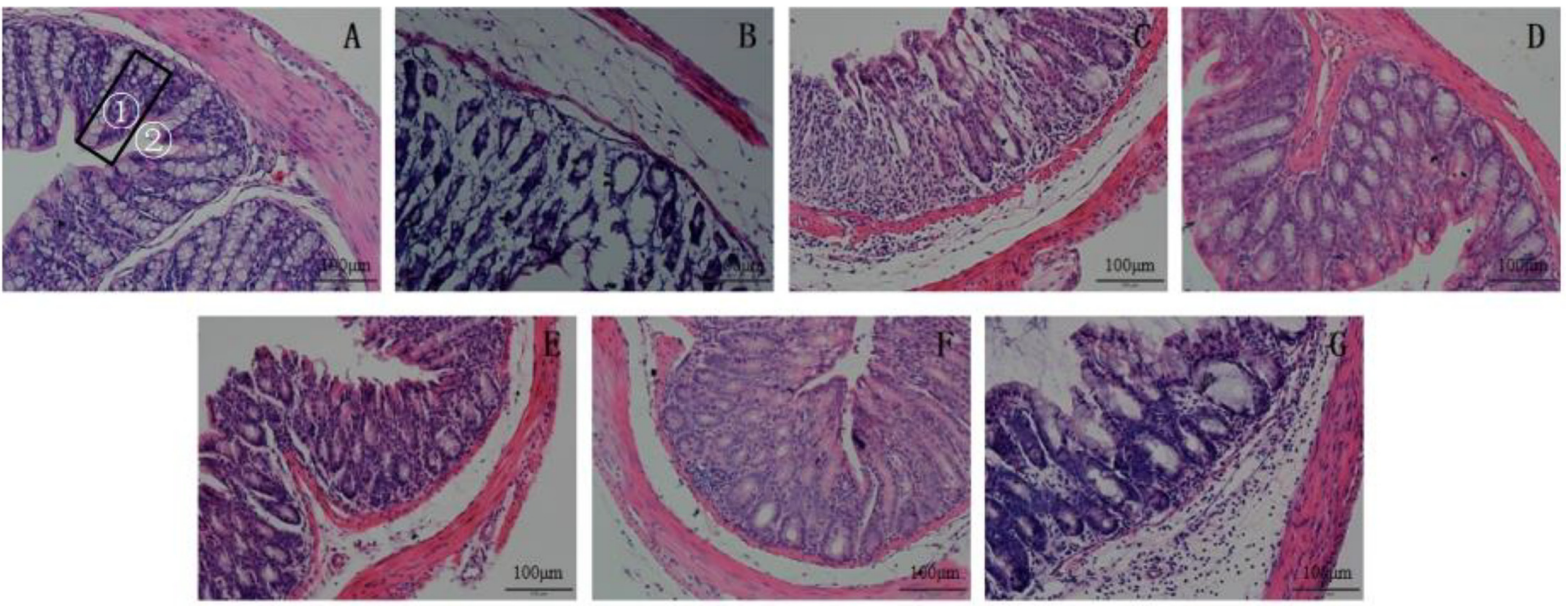
HE staining of mice colon sections. CON: PBS; Model: PBS+2.5%DSS; ATB: Antibiotic+2.5%DSS; PA: *P. acidilactici*+2.5%DSS; PA5I: *P. acidilactici*+Inulin (5 mg/ml)+2.5%DSS; PA10I: *P. acidilactici*+Inulin (10 mg/ml)+2.5%DSS; PA15I: *P. acidilactici*+Inulin (15 mg/ml)+2.5%DSS Note: (**A**) CON group (**B**) Model group (**C**) PA group (**D**) PA5I group (**E**) PA10I group (**F**) PA15I group (**G**) ATB group, scale: 100 μm ①: colonic villi ②: crypt

The impact of the combination of inulin and *P. acidilactici* on colon pathology in mice is depicted in Fig. 6. The CON group exhibited well-developed villi, normal crypt depth, intact gland structure, abundant goblet cells, and no infiltration of inflammatory cells, indicating a healthy state. In contrast, the Model group displayed significant loss of crypt and goblet cells along with severe damage to the mucosal and muscle layers due to infiltration by inflammatory cells; moreover, the serosal layer was completely absent. However, upon intragastric administration of inulin combined with *P. acidilactici*, inflammation was effectively alleviated across all treatment groups. Among them, the PA5I group exhibited the most remarkable amelioration in colonic pathological structural damage. Following seven days of DSS induction, a majority of epithelial structures retained their integrity, while improvements were observed in crypt and gland structure destruction, along with a significant reduction in inflammatory cell infiltration. The alleviating effects on the PA10I and PA15I groups ranked second-most prominent.

**Fig. 7:**
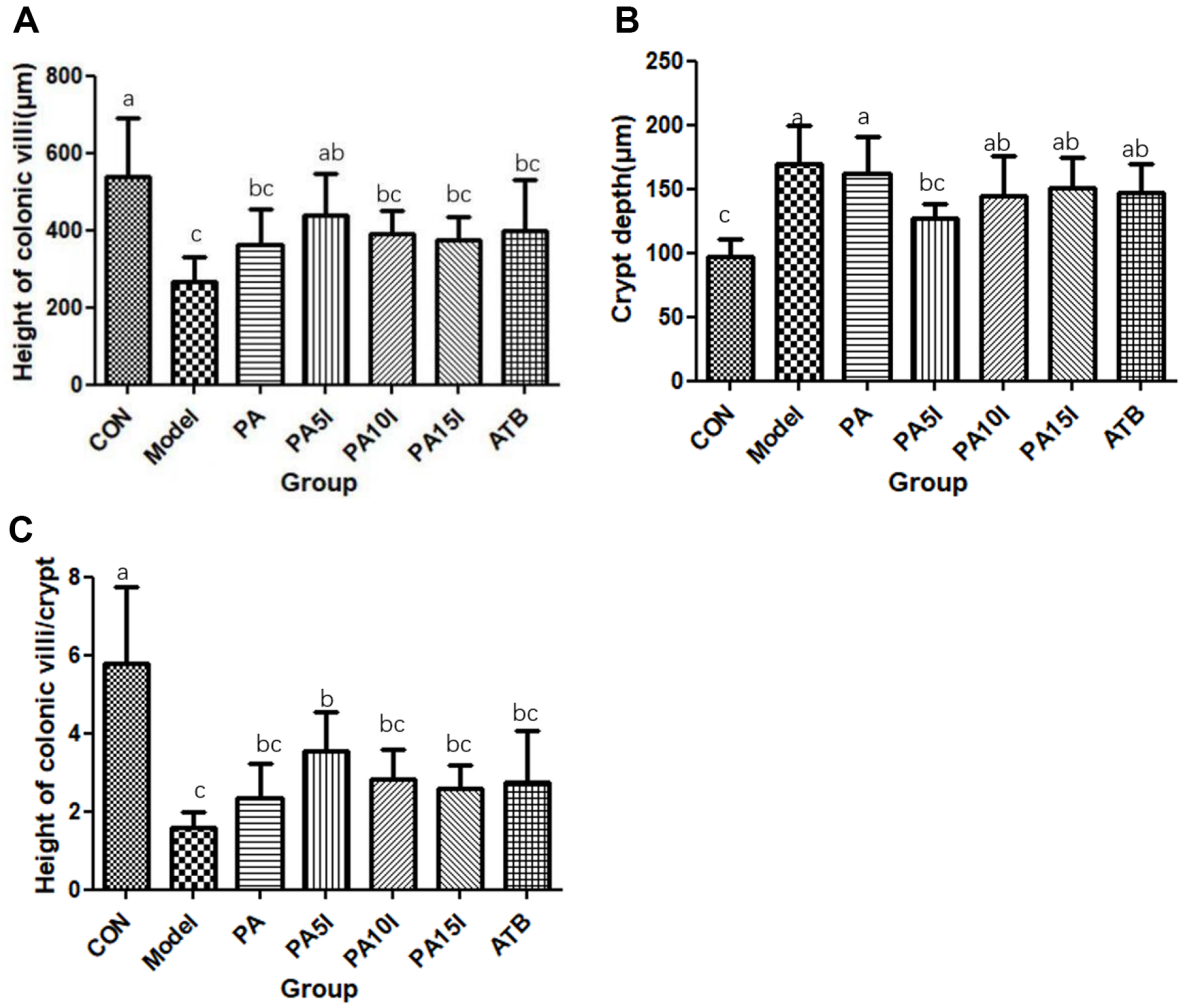
Changes of colonic villus height and crypt depth and their ratios in mice. CON: PBS; Model: PBS+2.5%DSS; ATB: Antibiotic+2.5%DSS; PA: *P. acidilactici*+2.5%DSS; PA5I: *P. acidilactici*+ Inulin (5 mg/ml)+2.5%DSS; PA10I: *P. acidilactici*+ Inulin (10 mg/ml)+2.5%DSS; PA15I: *P. acidilactici*+ Inulin (15 mg/ml)+2.5%DSS Note: (**A**) Height of colonic villi (**B**) Crypt depth (**C**) Height of colonic villi/Crypt depth Different letters above the histogram indicate significant differences (*p* < 0.05).

The effects of inulin combined with *P. acidilactici* on villus height, crypt depth, and their ratio in the murine colon are illustrated in Fig. 7A-7C. Statistical analysis revealed that the villus height was significantly higher (*p* < 0.05) in the PA5I group compared to the Model group, whereas the crypt depth was significantly lower (*p* < 0.05). Additionally, the ratio of villus height to crypt depth was significantly higher (*p* < 0.05) in the PA5I group than in the Model group. No significant differences were observed between the PA5I and CON groups regarding villus height or crypt depth (*p* > 0.05). These findings were further supported by our examination of pathological sections of colonic tissue, indicating that among all treatment groups, the PA5I group exhibited superior efficacy. Notably, the PA5I group demonstrated substantial improvement in terms of mitigating villus damage and crypt loss as well as alleviating inflammation within murine colon tissue.

### Effects on Oxidative Stress

The influence of inulin combined with *P. acidilactici* on oxidative stress-related indicators in mice is presented in Table 2. In comparison to the CON group, the Model group exhibited significantly elevated serum levels of MDA, MPO, and NOS (*p* < 0.05). Following intragastric intervention, excessive levels of MDA, MPO, and NOS were mitigated across all groups, with the ATB group demonstrating the most pronounced restorative effect (*p* < 0.05), while no significant difference was observed between the ATB group and CON group (*p* > 0.05). Regarding MDA levels, there was no notable distinction between the PA and PA5I groups when compared to the ATB group (*p* > 0.05), nor between these two groups and the CON group (*p* > 0.05). For MPO levels, there were no significant differences observed between the PA5I group and both ATB and CON groups (*p* > 0.05). Notably, as the inulin concentration decreased, so did its effectiveness gradually become more apparent.

The serum levels of SOD, GSH-Px, and T-AOC in the Model group were significantly lower compared to those in the CON group (*p* < 0.05). Following intragastric intervention, there was an increase in the levels of SOD, GSH-Px, and T-AOC in each treatment group. The ATB group exhibited the most pronounced recovery effect with no significant difference observed when compared to the CON group (*p* > 0.05). Additionally, there were no significant differences between the PA, PA5I, and PA10I groups compared to the ATB group (*p* > 0.05). As for inulin concentration reduction, a gradual enhancement of effects was observed.

In conclusion, there was a negative correlation between inulin concentration and efficacy, suggesting that the PA5I group may represent a promising alternative to antibiotics compared to other experimental groups.

### Effects on Serum Inflammatory Cytokine Levels

**Fig. 8:**
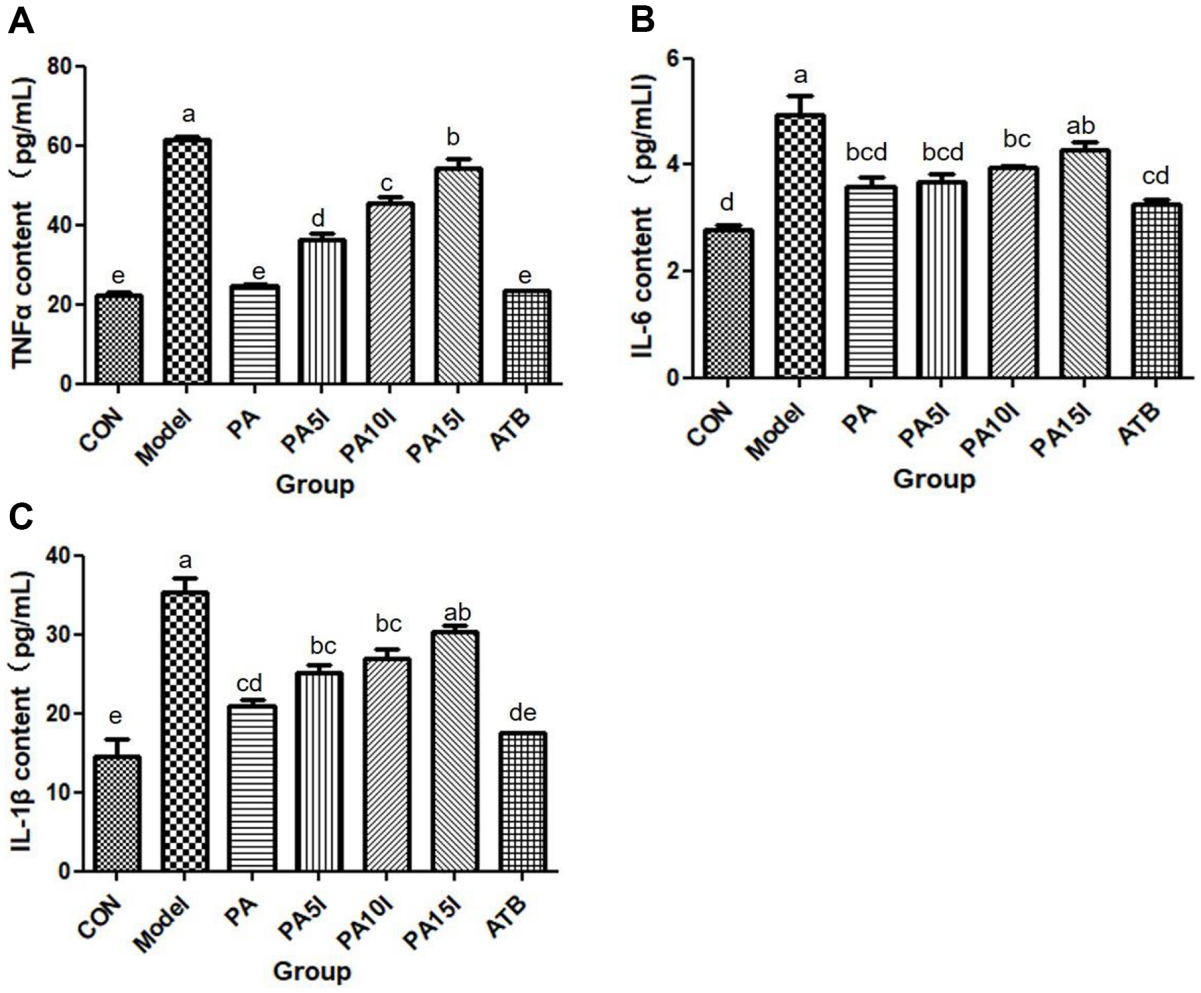
Effects of inulin combined with *Pediococcus acidilactici* on serum inflammatory cytokines in mice. CON: PBS; Model: PBS+2.5%DSS; ATB: Antibiotic+2.5%DSS; PA: *P. acidilactici*+2.5%DSS; PA5I: *P. acidilactici*+ Inulin (5 mg/ml)+2.5%DSS; PA10I: *P. acidilactici*+Inulin (10 mg/ml)+2.5%DSS; PA15I: *P. acidilactici*+Inulin (15 mg/ml)+2.5%DSS Note: (**A**)TNF-α levels in serum of mice, (**B**) IL-6 levels in serum of mice, (**C**) IL-1βlevels in serum of mice Different letters above the histogram indicate significant differences (*p* < 0.05).

The effects of inulin combined with *P. acidilactici* on serum inflammatory cytokine levels in mice are illustrated in Fig. 8. As depicted, the concentrations of serum TNF-α, IL-1β, and IL-6 were significantly elevated in the Model group compared to the CON group (*p* < 0.05). Following intragastric intervention using different administration methods, a decline in inflammatory cytokine levels was observed across all groups of mice. The level of TNF-α was notably lower than that of the Model group within each treatment group (*p* < 0.05), while no significant difference was found between the PA and CON groups (*p* > 0.05). With the exception of the PA15I group, all other treatment groups exhibited a substantial reduction in serum IL-6 content relative to the Model group (*p* < 0.05); no significant difference was observed between the PA and PA5I groups and the CON group (*p* > 0.05). Compared to the Model group, the PA15I group exhibited a significant decrease in serum IL-1β content (*p* < 0.05), while it was significantly increased compared to the CON group (*p* < 0.05). No significant differences were observed among the PA, PA5I, and PA10 groups (*p* > 0.05). These findings suggest that *P. acidilactici* and its combination with inulin can effectively ameliorate inflammation, particularly in the PA, PA5I, and PA10I groups.

## Discussion

Microecological agents, including probiotics, prebiotics, and synbiotics, play a crucial role in regulating intestinal flora. Probiotics have the ability to modulate the composition of gut microbiota, while prebiotics serve as a source of nutrients for probiotics [11]. Studies have demonstrated that synbiotics synthesized from the combination of probiotics and prebiotics can effectively promote intestinal microecological balance, alleviate colonic inflammation (IBD) in mice, enhance the abundance of beneficial bacteria in the intestine, and improve immune function [12, 13]. Whee-Soo Kima *et al*. [14] used the agar diffusion method to determine the antibacterial activity of PA cultured with 0.5% (w/v) PDN and 0.5% (w/v) dextran, respectively. The results showed that the inhibition zone of PA/PDN group was wider than that of PA/dextran group and PA group by agar diffusion test. Given that both inulin and dextran are prebiotic polysaccharides sharing similar properties, we selected *P. acidilactici* as the probiotic and inulin as the prebiotic for our synbiotics lavage intervention in mice. Subsequently, a colitis model was established by treating them with 2.5% DSS to investigate the efficacy of these synbiotics in alleviating colitis symptoms. Notably, significant improvements were observed in terms of body weight loss, increased DAI scores, colon shortening, spleen enlargement, and thymus atrophy following the administration of synbiotics via gavage intervention. Among these groups, PA5I exhibited the most remarkable improvement. In line with our findings, Chunyu Tian [15] also reported that continuous gavage of *L. casei* Q8-L and *L. paracasei* M5-L for 14 days had therapeutic effects on DSS-induced enteritis in mice and led to improved growth performance, consistent with our results.

Oxidative stress refers to the imbalance between the production and clearance of oxygen free radicals in the body or cells, leading to oxidative damage caused by the accumulation of reactive oxygen species (ROS) and reactive nitrogen species (RNS) [16]. Excessive free oxygen radicals can detrimentally affect cellular proteins, lipids, and nucleic acids. Extensive research has indicated a significant association between ROS/RNS and colitis pathogenesis. When oxidative stress occurs, tissue cells experience varying degrees of damage and destruction, particularly affecting lipid composition as well as amino acid and protein contents. In this experiment, DSS induction resulted in an overproduction of free radicals within mice bodies, compromising their antioxidant capacity and ultimately contributing to colitis development. Studies have demonstrated that enhancing the body's antioxidative ability can effectively prevent colitis onset.

The inﬂammatory response and oxidative stress are considered to be important pathological mechanisms that induce colitis, and the two processes mutually regulate and complement each other [17]. Oxidative stress plays a vital role in the pathogenesis of colitis, because the body’s own tissues can be attacked during the disease course. It can also induce inflammatory responses by generating pro-inflammatory substances via lipid peroxidation, leading to a sharp increase in inﬂammatory factors [18]. MDA is a lipid peroxidation product formed by the attack on unsaturated fatty acids (PUFA) in biofilms by oxygen free radicals produced by enzymatic and non-enzymatic systems [19], causing lipid peroxidation in the body [20], and its content can indirectly reflect the degree of cell damage caused by free radicals [21]. Further, MPO is mainly found in neutrophils, monocytes, and macrophages [22]. During inflammation, these cells accumulate at the site of inflammation and release MPO, causing further inflammatory changes [23]. The stronger the MPO activity, the more severe the inflammation in the body; therefore, it can also be used as an inflammatory indicator of oxidative stress [24]. NOS modulates cellular redox reactions by generating excessive nitric oxide (NO), playing a pivotal role in macrophages' initial inflammatory response upon pathogen invasion. The higher the NOS content, the stronger the inflammatory response [25]. SOD is an active substance distributed throughout the body [26]. As an antioxidant enzyme, SOD removes excess free radicals produced during metabolic processes, improves the body's immunity, and alleviates inflammation [27]. GSH-Px represents a peroxiredoxolytic enzyme capable of reducing bodily peroxides to prevent damage to cellular tissues. The intensity of its activity partially reflects resistance against colitis within the body [28]. T-AOC is the sum of the antioxidant capacities in the body [29]. The clinical biochemical examination index reflects the total ability of the body to remove ROS and RNS [30].

Changes in the levels of oxidative stress products and associated enzymes can induce inflammatory alterations within cells. In this experimental study, following synbiotic gavage intervention, a reduction in oxidative stress markers was observed across all groups compared to the Model group. Furthermore, there was a decrease in the levels of inflammation-related enzymes such as NOS and MPO, as well as the lipid peroxidation product MDA. Conversely, an increase in the activities of SOD, GSH-Px, and T-AOC was noted [31]. Notably, the most significant effect was observed in the PA5I group. Similar studies using probiotics and prebiotics to enhance antioxidant function in animals have been reported; Chen Ming *et al*. [32] used in vitro experiments to screen *Lactobacillus plantus* XM5 from yak yogurt on the Qinghai-Tibet Plateau, which has probiotic properties, to feed mice. The results showed that the activities of GSH-Px, SOD, and T-AOC in the serum of mice fed on *L. plantus* were significantly increased, whereas the MDA content in the serum decreased significantly [33]. Hui *et al*. [34] found that the inclusion of 1% *Lactobacillus*-fermented feed in the diet exhibited a significant enhancement in the activities of GSH-Px and SOD within the serum of growing pigs, while concurrently leading to a substantial reduction in MDA levels [35]. These findings align consistently with those observed in our present study.

In a murine model of DSS-induced colitis, the occurrence of colitis was directly associated with inflammatory cytokines. Elevated levels of these pro-inflammatory factors triggered an inflammatory cascade within the body, leading to severe colon damage and facilitating disease progression [36]. Among the inflammatory cytokines associated with ulcerative colitis (UC), TNF-α plays a pivotal role in initiating the inflammatory response, which manifests during the early stages of inflammation. TNF-α can be produced by activated macrophages or T cells, which in turn activate neutrophils to induce the release of other inflammation-related cytokines, thereby eliciting pro-inflammatory responses, such as enhanced vascular permeability and cellular necroptosis. The higher the content of TNF-α, the more severe the inflammation, so we used it as a crucial inflammation indicator and to detect the extent of the lesions. Previous studies have shown that TNF-α is a target for the treatment of IBD, and whether it can be effectively blocked or inhibited has become the key to relieve inflammation. Additionally, IL-6 and IL-1β are important proinflammatory factors in enteritis that exert pleiotropic effects by participating in immune defense, nerve cell function, and hematopoietic function. These cytokines also impact the growth and differentiation process of related cells, particularly T lymphocyte and B lymphocyte proliferation and differentiation [37]. Simultaneously, IL-1β promotes the pathological response of innate immunity by participating in the recruitment and retention of leukocytes in inflamed tissues. Under normal physiological conditions, the contents of IL-6 and IL-1β are usually maintained at a low level. However, during inflammatory pathological conditions, the immune response triggers antigen-activated immune cells to secrete substantial quantities of IL-1β and IL-6; thus, these cytokines can also serve as indicators for assessing the extent of inflammation [38]. Li *et al*.[39] demonstrated that rumen *Lactobacillus* exhibited an immunomodulatory effect on DSS-induced colitis in mice and effectively downregulated the levels of inflammatory cytokines [40]. In the present study, analysis of serum samples revealed significantly reduced levels of pro-inflammatory factors IL-6, IL-1β, and TNF-α in the synbiotics-treated group compared to the Model group. These findings highlight the potential of synbiotics with varying proportions of inulin to effectively alleviate intestinal inflammation in mice.

In conclusion, we investigated a synbiotic composed of inulin and *P. acidilactici* and evaluated its ability to alleviate dextran sodium sulfate (DSS)-induced colitis. Future studies will further investigate the preventive and therapeutic effects of inulin nanoparticles on colitis.

## Conclusion

This study demonstrated the beneficial effects of inulin combined with *P. acidilactici* synbiotics on growth performance, antioxidant activity, and colonic histological morphology in mice with DSS-induced colitis. Our findings provide experimental evidence to support further investigation into the efficacy of synbiotics for treating colitis.

## Figures and Tables

**Table 1:** DAI scoring criterion.

Score	Weight loss	Stool consistency	Degree of hematochezia
0	<1%	Normal	Normal
1	1-5%	Slightly loose but tangible	Mild occult blood
2	6-10%	Severely loose and unformed	Occult blood
3	11-15%	Loose stool	Hematochezia
4	>15%	Heavier loose stool	Severe hematochezia

**Table 2:** Regulatory effects of inulin combined with *Pediococcus acidilactici* on oxidative stress in mice.

Group	Item					
	GSH-PH(U/ml)	MDA(nmol/ml)	MPO(U/l)	NOS(U/ml)	SOD(U/ml)	T-AOC(U/ml)
CON	540.33 ± 24.71^a^	2.79 ± 0.14^d^	35.83 ± 2.43^c^	29.03 ± 1.98^d^	92.16 ± 13.97^a^	13.99 ± 1.26^a^
Model	348.56 ± 24.03^c^	4.93 ± 0.65^a^	61.07 ± 3.05^a^	53.33 ± 2.07^a^	64.29 ± 3.04^c^	7.38 ± 0.46^d^
PA	502.43 ± 7.53^ab^	3.58 ± 0.35^bcd^	55.57 ± 3.30^b^	42.06 ± 8.03^bc^	86.22 ± 0.85^ab^	12.74 ± 0.74^ab^
PA5I	471.25 ± 58.60^ab^	3.67 ± 0.28^bcd^	47.87 ± 1.84^bc^	37.84 ± 1.48^bc^	81.68 ± 2.42^ab^	11.61 ± 1.39^abc^
PA10I	454.25 ± 18.24^ab^	3.95 ± 0.03^bc^	52.85 ± 10.04^b^	45.81 ± 4.85^bc^	77.90 ± 3.86^abc^	10.70 ± 0.69^bc^
PA15I	435.08 ± 47.76^bc^	4.29 ± 0.25^ab^	57.66 ± 4.09^ab^	46.90 ± 1.93^b^	72.82 ± 3.72^bc^	9.88 ± 0.75^cd^
ATB	522.68 ± 3.38^ab^	3.25 ± 0.18^bcd^	42.31 ± 2.08^c^	34.02 ± 1.49^cd^	89.26 ± 2.13^ab^	13.31 ± 1.06^ab^

CON: PBS; Model: PBS+2.5%DSS; ATB: Antibiotic+2.5%DSS;

PA: *P. acidilactici*+2.5%DSS; PA5I: *P. acidilactici*+Inulin (5 mg/ml)+2.5%DSS;

PA10I: *P. acidilactici*+Inulin (10 mg/ml)+2.5%DSS;

PA15I: *P. acidilactici*+Inulin (15 mg/ml)+2.5%DSS

Note: (a) MDA content (b) MPO content (c) NOS content (d) SOD content

(e) GSH-PX content (f) TAOC content

Different letters above the histogram indicate significant differences (*p* < 0.05).

## References

[1] Das P, Horton R (2016). Antibiotics: achieving the balance between access and excess. Lancet (London, England).

[2] Spatz M, Da Costa G, Ventin-Holmberg R, Planchais J, Michaudel C, Wang Y (2023). Antibiotic treatment using amoxicillinclavulanic acid impairs gut mycobiota development through modification of the bacterial ecosystem. Microbiome.

[3] Kim PI, Jung MY, Chang YH, Kim S, Kim SJ, Park YH (2007). Probiotic properties of *Lactobacillus* and *Bifidobacterium* strains isolated from porcine gastrointestinal tract. Appl. Microbiol. Biotechnol..

[4] Kawakami SI, Yamada T, Nakanishi N (2010). Feeding of lactic acid bacteria and yeast on growth and diarrhea of holstein calves. J. Anim. Vet. Adv..

[5] Vaughn SE (2012). Review of the third edition of the guide for the care and use of agricultural animals in research and teaching. J. Am. Assoc. Lab. Anim. Sci..

[6] Kang XL (2018). Functional study of MicroRNA-5112 targeting IKKγ to regulate NF-κB inflammatory pathway [D].

[7] Liu X, Niu W, Liu JM, Cui Z, Li JZ, Zhang ZH (2023). Preparation of β-cyclodextrin and hydroxypropyl-β-cyclodextrin inclusion complexes of baicalein and evaluation of their effects on dextran sulfate sodium-induced acute ulcerative colitis in mice. J. Drug Deliv. Sci. Technol..

[8] Zhang LX, Yi HL (2022). Potential antitumor and anti-inflammatory activities of an extracellular polymeric substance (EPS) from *Bacillus subtilis* isolated from a housefly. Sci. Rep..

[9] Liu JW, Wang YH, Chen WC, Li S, Liu LF, Dang YH (2014). Subchronic exposure of apigenin induces hepatic oxidative stress in male rats. Health.

[10] Markowiak P, Śliżewska K (2018). The role of probiotics, prebiotics and synbiotics in animal nutrition. Gut Pathog..

[11] Maranduba CM, De Castro SB, de Souza GT, Rossato C, da Guia FC, Valente MA (2015). Intestinal microbiota as modulators of the immune system and neuroimmune system: impact on the host health and homeostasis. J. Immunol. Res..

[12] Wang YN, Meng XC, Dong YF, Zhao XH, Qian JM, Wang HY (2019). Effects of probiotics and prebiotics on intestinal microbiota in mice with acute colitis based on 16S rRNA gene sequencing. Chinese Med. J. Peking.

[13] Kim WS, Han GG, Hong L, Kang S K, Shokouhimehr M, Choi YJ (2019). Novel production of natural bacteriocin via internalization of dextran nanoparticles into probiotics [J]. Biomaterials.

[14] Tian CY (2018). Inhibitory effect of Lactobacillus on DSS-induced ulcerative colitis in mice [D].

[15] Huang Y (2019). Effect and mechanism of Alamandine on liver fibrosis by regulating oxidative stress and autophagy [D].

[16] Wang R, Luo Y, Lu Y, Wang DJ, Wang TY, Pu WY (2019). Maggot extracts alleviate inflammation and oxidative stress in acute experimental colitis via the activation of Nrf2. Oxid Med. Cell. Longev..

[17] Li F, Huang H, Zhu F, Zhou X, Yang Z, Zhao X (2021). A mixture of *Lactobacillus fermentum* HFY06 and arabinoxylan ameliorates dextran sulfate sodium-induced acute ulcerative colitis in mice. J. Inflamm. Res..

[18] Yang CB, Wang SK, Liu W, Ma ZY, Dou MM, Liu WT (2020). Anthocyanidin extract from summer-black-grape affects the expression of Ki-67 in testis, ovary of D-galactose-induced aging mice. J. Oleo Sci..

[19] Cho YI, Yoon KJ (2014). An overview of calf diarrhea - infectious etiology, diagnosis, and intervention. J. Vet. Sci..

[20] Wu HY (2008). Effects of heavy metals on antioxidative defense system and lipid peroxidation of Microcephalus obinatum [D].

[21] Lin JM (2013). Molecular mechanism of a Chinese family with complete deficiency of neutrophil MPO [D].

[22] Maldonado Galdeano C, Cazorla SI, Lemme Dumit JM, Vélez E, Perdigón G (2019). Beneficial effects of probiotic consumption on the immune system. Ann. Nutr. Metab..

[23] Li CW (2014). Chemical composition analysis and anti-inflammatory activity evaluation of ethanol extract of underground part of patchouli rugosa [D].

[24] Rong XL (2020). The effect of ginger on ulcerative colitis and intestinal flora in mice induced by dextran sodium sulfate [D].

[25] Li XN (2011). Study on extraction and separation of antioxidant components of Camellia oleiferae seed shell [D].

[26] Tsuruta T, Katsumata E, Mizote A, Jian HJ, Muhomah TA, Nishino N (2020). Cyclic nigerosylnigerose ameliorates DSS-induced colitis with restoration of goblet cell number and increase in IgA reactivity against gut microbiota in mice. Biosci. Microbiota Food Health.

[27] Kim JJ, Shajib MS, Manocha MM, Khan WI (2012). Investigating intestinal inflammation in DSS-induced model of IBD. J. Vis. Exp..

[28] Xi XJ (2018). Clinical significance of imbalance between oxidation and antioxidant in saliva of patients with chronic obstructive pulmonary disease [D].

[29] Muralidhara KS, Sheggeby GG, Elliker PR, England DC, Sandine WE (1977). Effect of feeding Lactobacilli on the coliform and *Lactobacillus Flora* of intestinal tissue and feces from piglets. J. Food Prot..

[30] Li WW, Wang JY, Chen ZQ, Gao XD, Chen Y, Xue ZH (2018). Physicochemical properties of polysaccharides from Lentinus edodes under high pressure cooking treatment and its enhanced anticancer effects. Int. J. Biol. Macromol..

[31] Chen M, Ke WC, Zhang J, Tang J, Wang LN, Ding WR (2017). In vitro and in vivo probiotic properties of lactic acid bacteria with antioxidant activity in yak yogurt of Qinghai-Tibet plateau. Food Sci..

[32] Zhou XR, Tan Q, Mu JF, Zeng S, Zeng YT, Zhao X (2020). Isolation and screening of lactic acid bacteria from pickles and its improvement of oxidative stress level in mice. Modern Food Sci. Technol..

[33] Liu H, Ji HF, Wang SX, Zhang DY, Wang J, Zhang W (2021). Effects of compound *Lactobacillus* fermented feed on growth performance, fecal flora, serum immune and antioxidant Indexes of growing pigs. Chin. J. Anim. Nutr..

[34] Jin SJ (2018). Effects of compound microbial fermentation broth on growth performance, serum indexes and fecal VFA of weaned piglets [D].

[35] Illippangama AU, Jayasena DD, Jo C, Mudannayake DC (2022). Inulin as a functional ingredient and their applications in meat products. Carbohydr. Polym..

[36] Sun NX, Teng AG, Zhao YN, Liu HP, Tu JQ, Jia Q (2020). Immunological and anticancer activities of seleno-ovalbumin (Se-OVA) on H22-bearing mice. Int. J. Biol. Macromol..

[37] Xu XF (2021). Therapeutic effect and mechanism of berberine on dextran sodium sulfate induced colitis in mice [D].

[38] Li MJ, Yang B, Wang S, Zhao JX, Zhang H, Chen W (2021). Lactobacillus ruminosus ameliorates DSS-induced colitis by regulating inflammatory cytokines and intestinal flora [C]//.

[39] Yu W (2019). Pharmacodynamics and mechanism of dimethylsulfone screened from urine of COPD patients and healthy volunteers [D].

